# Supplementary material for: Deciphering TAL effectors for 5-methylcytosine and 5-hydroxymethylcytosine recognition
Source: Nat Commun. 2017 Oct 12;8:901. doi: 10.1038/s41467-017-00860-6 (PMC5638953; doi:10.1038/s41467-017-00860-6)
Supplement: Supplementary file 1 — Supplementary Information [file 41467_2017_860_MOESM1_ESM.pdf]

Supplementary Figures

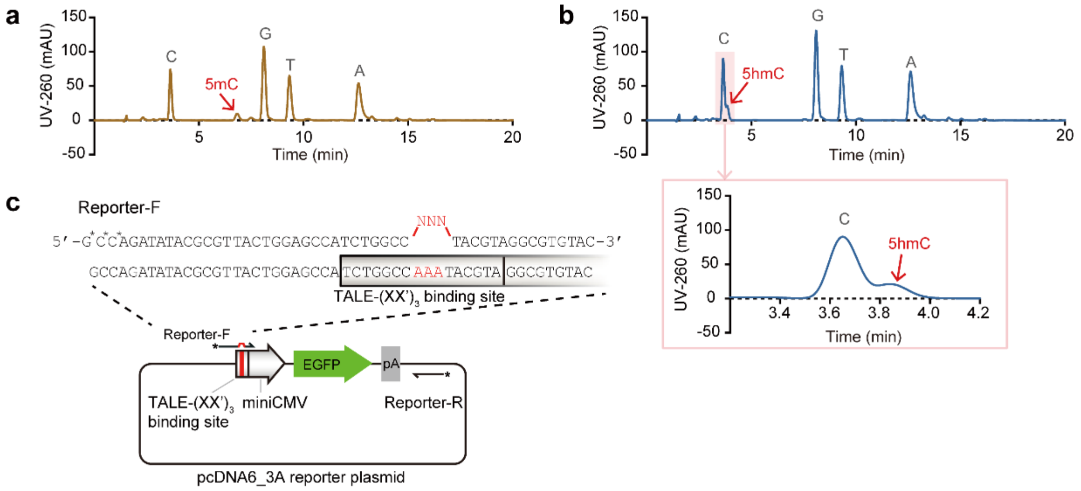

Supplementary Figure 1. Preparation of 5mC and 5hmC containing reporters. (**a and b**) 5mC and 5hmC was incorporated into the primer used for generating 5mC and 5hmC containing reporters. The HPLC chromatographs showed the incorporation of 5mC (**a**) and 5hmC (**b**); a zoom-in picture is showed for clear observation of 5hmC peak. (**c**) Schematic of PCR amplification of 5mC and 5hmC containing reporters.

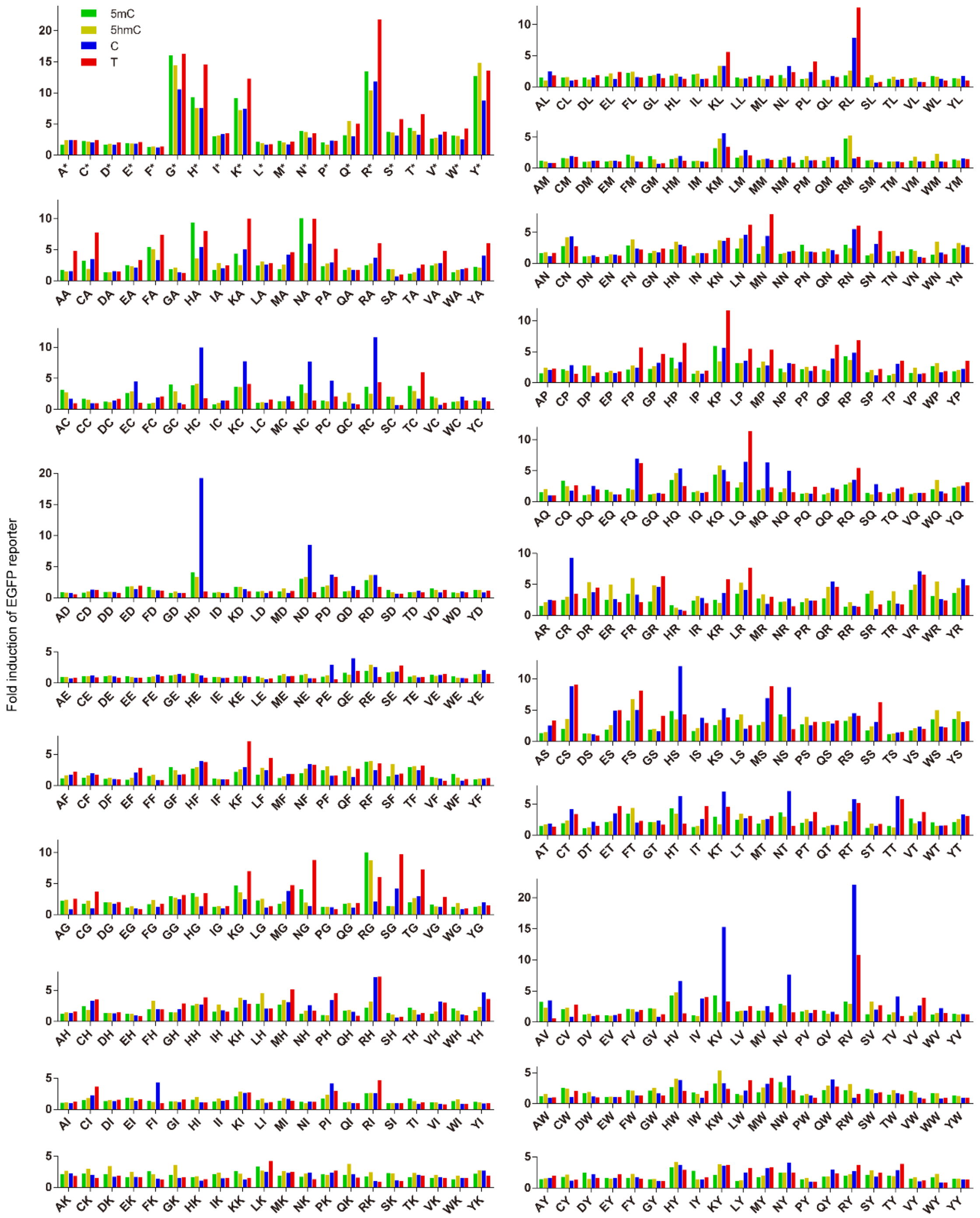

Supplementary Figure 2. Binding preferences of 420 TALE RVDs to modified cytosines. The data is corresponding to that of the heat map (Fig. 2a). The Y-axis is fold induction of EGFP reporter, and the X-axis is the RVDs. The bar plot is categorized by the first residue of RVD, and the data is listed according to the second residue alphabetically.

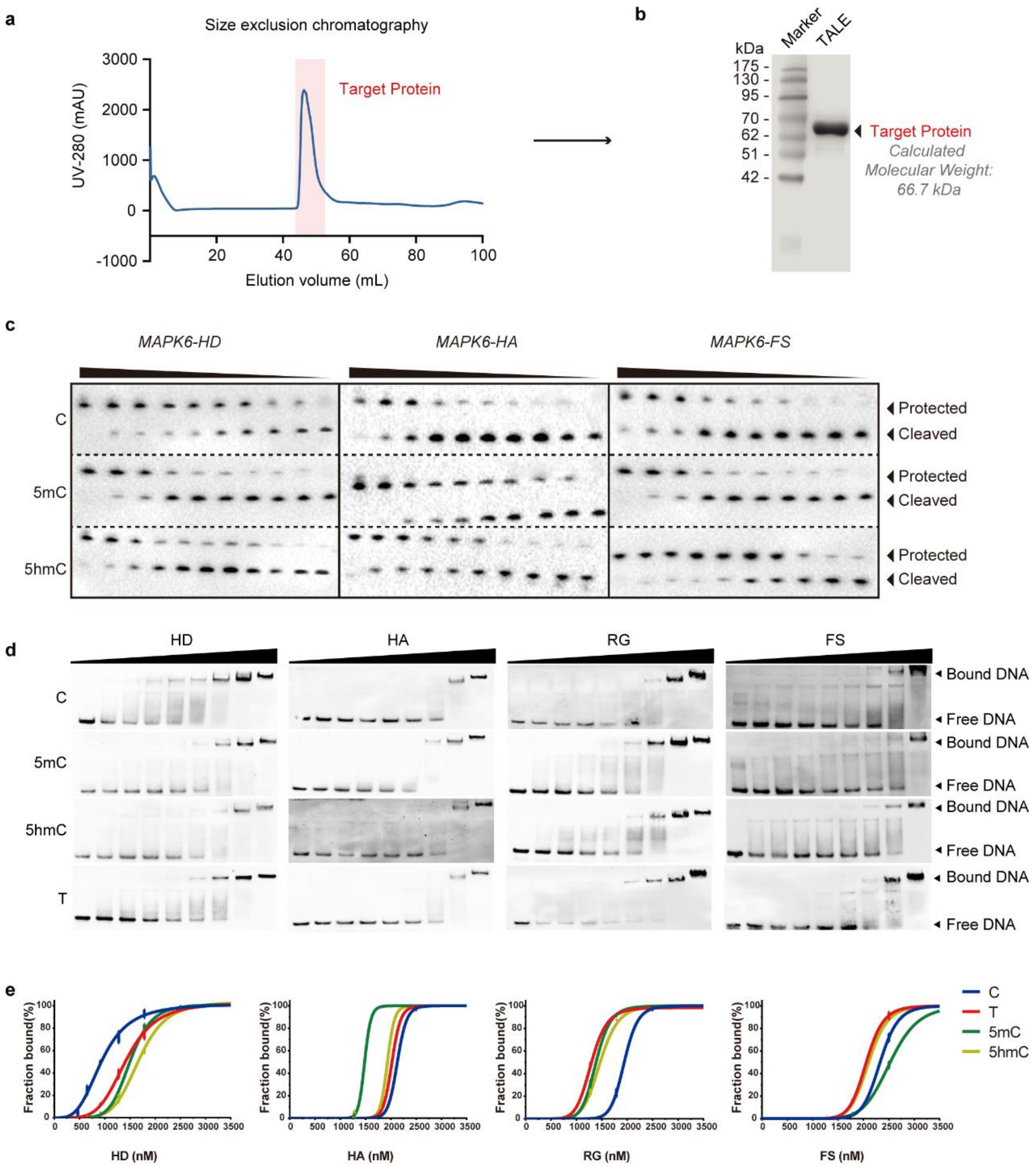

Supplementary Figure 3. Specific binding of epigenetic cytosines and T by variant TALE RVDs *in vitro*. (**a**) A representative size exclusion chromatography of purified TALE proteins. (**b**) SDS-PAGE analysis showed the molecular weight of purified TALE protein correlates well with the calculated molecular weight. (**c**) Representative gel images of *in vitro* protection assay. *MAPK6*-HD could protect C with the highest efficiency, while HA protected 5mC and 5hmC with higher efficiency than that of unmodified C and FS protected 5hmC with the highest efficiency. (**d**) Representative gel images of electrophoretic mobility shift assay. HD binds strongly to C but poorly to 5mC, 5hmC and T; HA specifically recognizes 5mC but not other cytosines or T; RG binds well to both 5mC, 5hmC and T but not C; FS preferentially recognizes 5hmC and T than 5mC and C. (**e**) The different TALE RVDs binding efficiency curves of (**d**), as measured by the fraction of the bound DNA. The curves are fitted into Specific Binding curve with Hill slope (GraphPad). Data are means ± s.d., n = 2; *P < 0.05, and **P < 0.005.

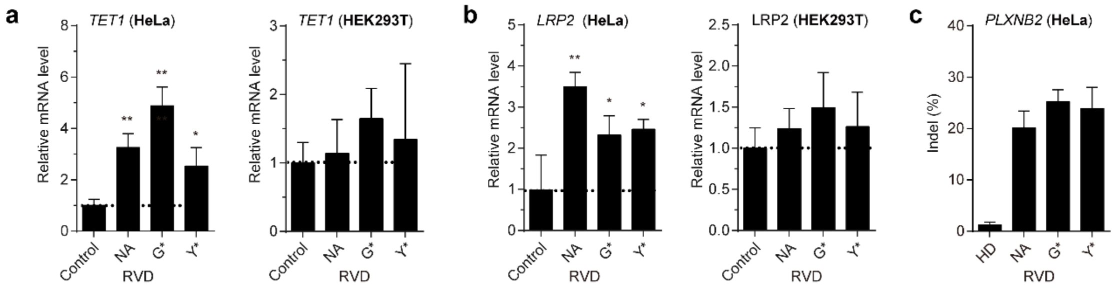

Supplementary Figure 4. Methylation-dependent gene expression activation and genome editing. (**a**) The relative mRNA level of *TET1* in HeLa and HEK293T cells transfected with TALE$_{TET1}$ with RVDs NA, G* and Y*. (**b**) The relative mRNA level of *LRP2* in HeLa and HEK293T cells transfected with TALE$_{LRP2}$ with RVDs NA, G* and Y*. (**c**)The genome editing efficiency of TALEN with RVDs NA, G* and Y*. Data are means ± s.d., n = 3; *P < 0.05, and **P < 0.005.

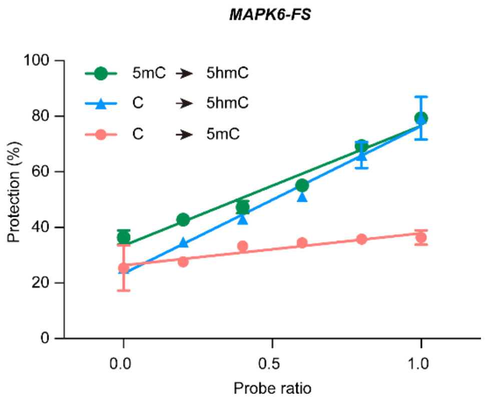

Supplementary Figure 5. Selective protection of 5hmC-containing DNA by TALE-FS. 5mC, 5hmC and unmodified C containing DNA (with same sequence from *MAPK6* gene) were pairwise mixed in different proportions. As the fraction of 5mC (red) increased, the protection efficiency only slightly increased. As the fraction of 5hmC increased (mixed with C and 5mC, green and blue), the protection efficiency greatly increased, indicating the selective protection of 5hmC using RVD FS.
